# From disease modelling to personalised therapy in patients with *CEP290* mutations

**DOI:** 10.12688/f1000research.11553.1

**Published:** 2017-05-12

**Authors:** Elisa Molinari, Shalabh Srivastava, John A. Sayer, Simon A. Ramsbottom

**Affiliations:** 1 Institute of Genetic Medicine, Newcastle University, Newcastle, NE1 3BZ, UK; 2 Renal Services, Newcastle upon Tyne NHS Foundation Trust, Newcastle, NE7 7DN, UK

**Keywords:** Cep290, splicing, genetic pleiotropy, exon-skipping, Leber congential amaurosis, Joubert syndrome, nephronophthisis

## Abstract

Mutations that give rise to premature termination codons are a common cause of inherited genetic diseases. When transcripts containing these changes are generated, they are usually rapidly removed by the cell through the process of nonsense-mediated decay. Here we discuss observed changes in transcripts of the centrosomal protein CEP290 resulting not from degradation, but from changes in exon usage. We also comment on a landmark paper (Drivas *et al*. Sci Transl Med. 2015) where modelling this process of exon usage may be used to predict disease severity in *CEP290* ciliopathies, and how understanding this process may potentially be used for therapeutic benefit in the future.

## Mechanisms of aberrant transcript removal

Nonsense mutations are changes to the coding sequence of a gene, which lead to a termination (stop) codon being coded for in place of an amino acid, giving rise to a truncated protein. These types of mutations are common in human disease and account for around a third of inherited genetic disorders ^1^. The resultant abnormally truncated proteins can have significant deleterious effects on the cell. A truncated protein may display a loss-of-function or in some cases a dominant-negative function that can seriously impact on normal biological processes. Two mechanisms have been identified by which cells remove unwanted transcripts, thereby avoiding production of abnormal protein. The first of these is nonsense-mediated decay (NMD), whereby transcripts containing a nonsense mutation are targeted for degradation giving rise to a reduction in transcript ^2, 3^. The second method that has been described is that of nonsense-associated altered splicing (NAS), a mechanism that promotes the increase in transcripts that are missing the exon containing the deleterious mutation ^4^.

Many genes are alternately spliced in order to generate proteins with unique properties and functions ^5^. However, NAS, triggered by the presence of a premature stop codon, leads to splicing of an exon that may not normally be spliced. Evidence of NAS has been shown following mutation of *CEP290* ^6^, a gene that, when mutated, is associated with a spectrum of inherited genetic disorders, including Leber congenital amaurosis (LCA), Senior Løken syndrome (SLS), Joubert syndrome (JBTS) and Meckel-Gruber syndrome ^7– 10^. The compound heterozygous mutations described in a family with LCA by Littink *et al*. included a novel premature termination codon in exon 7 (c.451C>T, p.Arg151*). When mRNA transcripts were sequenced, skipping of either exon 7 alone, or exon 7 and exon 8 was revealed, which was never seen in controls ^6^. The authors concluded that the LCA phenotype seen in the patient, which was less severe than expected, was due to a functional CEP290 protein being produced due to NAS. The genetic pleiotropy exhibited in patients with *CEP290* mutations may therefore be explained in part by the differential ability of NAS to give rise to a functional protein in the event of a premature termination codon being generated. One can surmise that the greater the level of near-normal (wild-type) protein that can be generated, the lower the disease burden and the milder the phenotype.

## Predicting disease severity using alternate splicing models

The recent landmark article by Drivas *et al.* has provided some novel insights into the potential modelling of *CE290* mutations, based around the idea that genetic pleiotropy may arise as a result of differences in protein levels ^11^. Known mutations in *CEP290* were classified into categories based on the premise that the severity of disease correlates with the impact on the overall level of functional protein that may be generated. Missense mutations were classified as mild, as they should impact less on the level of transcript, whereas nonsense mutations were either moderate or severe depending on whether or not they occur in a codon that begins and ends in the same frame and can therefore be spliced out with no change in reading frame. In theory, transcripts containing nonsense mutations would be removed by NMD, so the overall level of *CEP290* transcript would be lower in those patients harbouring mutations in exons that may not be easily skipped by NAS. Using this simple model, it was shown that the disease severity does correlate fairly well with the predicted level of CEP290 protein. The authors then modified the model to take into account skipping of regions of known functional importance. Mutations that map to these regions have more severe phenotypes than can be explained by the original model, due to the fact that skipping out these exons gives rise to a protein with reduced functionality.

When the model was tested against genotype-phenotype correlations in patients with varying symptoms, it did appear to accurately predict the phenotype from the given genotype ^11^. Furthermore, the authors showed that for mutations that give rise to a premature termination codon, the exon in which the mutation has occurred is indeed spliced out, as would be expected if NAS were activated. However, what was unexpected was that in control samples these exons were also shown to be spliced out. Perhaps even more surprisingly, the spliced levels observed in control samples were a similar level to that of the patient samples. The fact that the levels observed in controls are the same as seen in patients suggests that it is in fact not NAS that is leading to splicing of these exons; splicing is simply happening at a basal level and is not in any way increased due to the presence of the mutation.

Importantly, while it was shown by PCR that small amounts of these transcripts existed, the authors were unable to detect transcripts arising from basal exon skipping via direct RNA-sequencing, which suggests the infrequent nature of these alternative splicing events and so brings into question the biological relevance of this mechanism. It must also be noted that there are patients who have the same or similar mutations, but present with symptoms of differing severity. One reported *CEP290* mutation (c.21G>T; p.Trp7Cys) gives rise to both SLS and JBTS phenotypes ^8, 9^. This mutation is in exon 2, which contains the start codon, and so may not be spliced out. In this case, the genotype alone is unable to be used to predict how the transcript level will impact on the severity of the disease and is therefore no use as a proxy measure for phenotype. Similarly, there have been several patients reported with a nonsense mutation in exon 36 (c.4723A>T; p.1575*), who present with LCA and not JBTS ^12– 14^, even though this mutation is only a few bases upstream of the c.4732G>T; p.Glu1578* mutation with JBTS phenotypes ^8^. It must therefore be acknowledged that exon skipping is not the sole source of genetic pleiotropy.

## Manipulation of splicing for therapeutic benefit

Patients suffering from non-syndromic LCA commonly have a mutation within the *CEP290* gene (c.2991+1655A>G), which creates a cryptic splice site, resulting in the inclusion of an aberrant exon of 128 bp that contains a premature stop codon (p.Cys998*). Alternative splicing of the cryptic exon occurs in some, but not all, mRNA transcripts ^15, 16^. Collin *et al*. successfully exploited the use of antisense oligonucleotides (AONs) to boost an efficient skipping of the mutant cryptic exon: by transfecting AONs in patient-derived lymphoblastoid cells, they were able to redirect normal splicing of *CEP290* ^17^. As a proof of principle for the feasibility of altering the splicing pattern of *Cep290 in vivo* in the affected tissue, intravitreal injection of wild type mice with naked splice-switching AON led to the modification of *Cep290* splicing in retinal cells ^18^. Similarly, naked AONs and adeno-associated virus-packaged AONs were administered to a humanized mouse model ( *Cep290 ^lca/lca^*) that contains intron 26 of the human *CEP290* gene carrying the c.2991+1655A>G mutation. Delivery by intraocular injection caused a statistically significant reduction of aberrantly spliced *Cep290* up to 1 month after injection, without compromising the retinal structure ^19^. However, humanized *Cep290 ^lca/lca^* mouse fails to recapitulate the human clinical features, making it impossible to understand the actual impact of AON-directed restoration of wild type *Cep290* transcript on the retinal phenotype ^18– 20^. Nevertheless, the ability of splice-switching AONs not only to cause an upregulation of wild type *CEP290* mRNA to normal levels, but also to restore otherwise impaired ciliogenesis in patient-derived fibroblast cells, demonstrates, although only in a limited way, that an increase of correctly spliced transcript can indeed result in a phenotypical rescue ^21^.

Using a similar approach, AONs can be exploited to promote skipping of exons carrying nonsense mutations to increase the abundance of slightly shortened transcripts and near-full length functional protein, as in the case of mutated dystrophin in Duchenne muscular dystrophy ^22^.

The majority of *CEP290* mutations are nonsense mutations that introduce a premature stop codon in the mRNA ^9^. In addition to retinal degeneration, these mutations cause a wide spectrum of multisystemic ciliopathies, such as the cystic kidney disease nephronophthisis, which results in end stage renal failure at a median age of 13 years. Due to the relatively slow progression of this disease, there is a potential time for therapeutic intervention. If CEP290 protein levels could be restored by inducing exon skipping, disease progression may be significantly slowed or even halted. As we move closer to personalised medicine, especially in the arena of rare disease, it is likely that therapeutic strategies such as this may become routine, with unique therapies being designed based on the patient’s genotype. Understanding the way in which deleterious mutations are dealt with *in vivo* will have a significant impact on how successfully these therapies can be implemented.
