# Supplementary material for: From disease modelling to personalised therapy in patients with CEP290 mutations
Source: F1000Res. 2017 May 12;6:669. [Version 1] doi: 10.12688/f1000research.11553.1 (PMC5482330; doi:10.12688/f1000research.11553.1)
Supplement: Peer review history

# Peer review: Referee response for version 1

**DOI:** 10.5256/f1000research.12479.r23067

**Published:** 2017-06-19

**Authors:** Ruxandra Bachmann-Gagescu

**Affiliations:** 1 Institute for Molecular Life Sciences, Institute of Medical Genetics, University of Zurich, Zürich, Switzerland

The correspondence article by Molinari and colleagues discusses potential therapeutic options through manipulation of splicing for patients harbouring *CEP290* mutations. This represents an important topic given the role of *CEP290* in human disease and the intense research focusing on understanding the phenotypic consequences of *CEP290* dysfunction and identifying therapies for affected individuals. Thus the rationale for writing this correspondence article is clearly established.

 The article by Molinari and colleagues reviews various studies that have provided a possible explanation to the important phenotypic pleiotropy caused by *CEP290* mutations through exon skipping to by-pass stop mutations (“non-sense associated altered splicing”). In particular, they thoroughly comment on the paper by Drivas *et al.* who developed an algorithm to predict phenotypic severity based on the location of the mutation within the *CEP290* gene and consequent anticipated CEP290 protein levels. After discussing this model in depth, Molinari and colleagues suggest that targeted modulation of splicing through antisense oligonucleotide-based therapy is an interesting therapeutic option for patients with *CEP290* mutations.

 The opinions expressed in this correspondence article are well balanced and supported by appropriate citations. The authors thoroughly comment on the study by Drivas *et al*, highlighting strengths but also discussing limitations. In particular, they comment on the fact that exon skipping appears to mostly occur not through “nonsense-associated altered splicing” as expected but rather through “basal exon skipping” independently of the presence of a mutation. While this finding questions the relevance of exon skipping occurring *in vivo* in the presence of stop mutations, it would probably not question the feasibility of splicing modulation as a therapeutic modality; increasing the proportion of transcript by-passing the mutation should partially rescue protein function, regardless of whether such a mechanism is triggered *in vivo* to attenuate consequences of a mutations on protein function.

 Besides these considerations, one important point that is not addressed in the work by Drivas *et al*, but that is appropriately raised by Molinari and colleagues, is the fact that phenotypic differences are observed even between individuals sharing the same causal mutations, indicating that other mechanisms must influence the phenotypic outcome. Similarly, our recent work on a large JS cohort (Phelps *et al*, *Genetics in Medicine* in press) noted phenotypic discrepancies despite identical causal mutations in 60% of situations. Therefore, while the causal mutations certainly play a major role in determining the phenotypic outcome, additional mechanisms must also underlie the genetic pleiotropy observed.

 The discussion by Molinari and colleagues could have been expanded to include additional points:

- In commenting on the work by Drivas *et al,* it should be pointed out that the correlation between the anticipated protein levels and the phenotypic severity is strongly influenced by the phenotypic assessment. The study by Drivas *et al* mostly relied on phenotypic descriptions from the literature and misclassification of patients (as having JS instead of JSRD for example, if no thorough assessments of retinal or renal function were performed) cannot be ruled out. Moreover, determining the “phenotypic severity” is more difficult than it seems. The commonly used scale considers LCA as the least severe disease manifestation, MKS as the most severe and JS with or without additional features in-between. However, one can argue whether single-organ involvement resulting in blindness in early childhood (LCA) is really less severe than presence of the molar tooth sign with mild developmental delay (classified as JS) or whether JS with mild developmental delay and polydactyly (classified as JSRD) is more severe than JS with severe developmental delay but no additional organ system involvement (classified as JS). From a biological standpoint, it remains yet to be demonstrated that the degree of dysfunction of a given protein is necessarily correlated with the number of tissues affected. One could provide alternative hypotheses involving the presence of additional variants in other genes (modifiers) or suggesting that different regions of the protein are important for different functions in different cell types. These points might affect the reliability of the prediction algorithm proposed by Drivas and colleagues which in turn would question the efficiency of inducing exon skipping to decrease phenotypic severity in patients. Larger studies relying on thorough standardized phenotypic assessments as performed for 6 patients in the study by Drivas *et al.* and a more nuanced classification of phenotypic severity would be helpful in confirming the reliability of the proposed exon-skipping model in predicting phenotypic outcome.
- Additional issues should also be considered when thinking about controlled exon skipping as a therapeutic modality. Exon skipping may not rescue the phenotype as efficiently in genes such as *CC2D2A*, in which missense mutations predominate in JS/JSRD. Finally, splicing modulators may be challenging to apply in patients harbouring compound heterozygous mutations in different exons of the target gene as each splicing modulator can affect both alleles, resulting in transcripts missing multiple exons.

These points may represent limitations in the applicability or efficiency of splicing-modulation as a therapeutic option which could have been discussed by Molinari and colleagues. Nevertheless, as appropriately discussed by the authors, this avenue deserves further investigation and *CEP290* is certainly the best candidate among the ciliopathy genes for such type of intervention given the high proportion of truncating mutations and the progressive nature of retinal and renal complications providing a time window for intervention.

I have read this submission. I believe that I have an appropriate level of expertise to confirm that it is of an acceptable scientific standard.

# Peer review: Referee response for version 1

**DOI:** 10.5256/f1000research.12479.r23066

**Published:** 2017-06-19

**Authors:** Patricia D. Wilson

**Affiliations:** 1 Centre for Nephrology, UCL Medical School, University College London, London, UK

This clearly-written and well-argued manuscript highlights recent pertinent findings concerning mechanisms underlying exon skipping and splicing in determination of disease severity in patients with CEP290 mutations. Evidence cited is consistent with the notion that the severity of disease is inversely proportional to the level of normal or near-normal protein generated. It is suggested that phenotypic rescue, could be achieved in patients with the most common, severe nonsense mutations of CEP290 by in-frame splicing. This has important potential for personalised therapeutic intervention in the future which envisages induction of exon skipping to up regulate normal mRNA, increase near-normal protein levels and thereby reduce the severity and progression of for instance nephronopthisis, a rare disease but common cause of renal failure in children.

I have read this submission. I believe that I have an appropriate level of expertise to confirm that it is of an acceptable scientific standard.
